# Shock-Induced Energy Release Performances of PTFE/Al/Oxide

**DOI:** 10.3390/ma15093042

**Published:** 2022-04-22

**Authors:** Ying Yuan, Dongfang Shi, Suo He, Huanguo Guo, Yuanfeng Zheng, Yong Zhang, Haifu Wang

**Affiliations:** Beijing Institute of Technology, Beijing 100081, China; 3120185181@bit.edu.cn (Y.Y.); 3120210210@bit.edu.cn (D.S.); hesuo@bit.edu.cn (S.H.); 7520200026@bit.edu.cn (H.G.); zhengyf@bit.edu.cn (Y.Z.); 1120193567@bit.edu.cn (Y.Z.)

**Keywords:** PTFE/Al/oxide, energy release performance, pressurization characteristics, shock-induced

## Abstract

In recent years, polytetrafluoroethylene (PTFE)/aluminum (Al) energetic materials with high-energy density have attracted extensive attention and have broad application prospects, but the low-energy release efficiency restricts their application. In this paper, oxide, bismuth trioxide (Bi_2_O_3_) or molybdenum trioxide (MoO_3_) are introduced into PTFE/Al to improve the chemical reaction performance of energetic materials. The pressurization characteristics of PTFE/Al/oxide as pressure generators are compared and analyzed. The experiments show that the significantly optimized quasi-static pressure peak, impulse, and energy release efficiency (0.162 MPa, 10.177 s·kPa, and 0.74) are achieved for PTFE/Al by adding 30 wt.% Bi_2_O_3_. On the other hand, the optimal parameter obtained by adding 10% MoO_3_ is 0.147 MPa, 9.184 s·kPa, and 0.68. Further, the mechanism of enhancing the energy release performance of PTFE/Al through oxide is revealed. The mechanism analysis shows that the shock-induced energy release performance of PTFE/Al energetic material is affected by the intensity of the shock wave and the chemical reaction extent of the material under the corresponding intensity. The oxide to PTFE/Al increases the intensity of the shock wave in the material, but the chemical reaction extent of the material decreases under the corresponding intensity.

## 1. Introduction

Polytetrafluoroethylene (PTFE)/aluminum (Al) is a class of energetic materials, which have great application potential in weapon damage and propellants. Many studies have been conducted on PTFE/Al, mainly including the mechanics properties [1] and reaction characteristics [2,3,4,5]. In spite of PTFE/Al having unique characteristics, such as a high energy density (21 kJ/cm^3^) [6], ease of molding, and the release of energy under highly dynamic loads, how to optimize its energy release performance is still a great challenge, which limits the applications.

Many components have been added to PTFE/Al to control the chemical reaction, enhancing the energy release performance. The added components mainly include three types: self-reactive energetic materials, such as TiH_2_ [7]; fuels reacting with PTFE, such as Mg [8]; oxidizing agents reacting with Al, such as a metal oxide. Among them, metal oxides are regarded as the most promising additive to PTFE/Al-based energetic material, and some studies using them have been conducted. The shock-induced energy release characteristics of PTFE/Al/CuO energetic materials have been studied [9] and the experiments indicated that CuO improves the energy release efficiency. The reaction process at different temperatures for PTFE/Al/MnO_2_ has been analyzed by DSC/TG-MG and XRD techniques [10]. The study indicated that PTFE/Al/MnO_2_ presents a shorter reaction time, a faster energy release rate, and a better heat release performance. The shear-induced initiation mechanism of PTFE/Al/MoO_3_ was revealed based on compression experiments, and the reaction products were AlF_3_, Al_2_O_3_, Mo, and C [11]. The impact reaction characteristics and impact sensitivity of PTFE/Al/Bi_2_O_3_ were investigated with a different Bi_2_O_3_ content, and it showed that with a Bi_2_O_3_ content increasing from 0 to 35.616%, the impact sensitivity first increased and then decreased [12]. The reaction characteristics of PTFE/Al/oxide, in which the oxide involved was Bi_2_O_3_, MoO_3_, and Fe_2_O_3_, were investigated and compared, and the experiments showed that Bi_2_O_3_ was the most effective oxidant in increasing the reaction efficiency of the PTFE/Al system [13].

However, the mechanism of the effect of oxide content on the shock-induced energy release of PTFE/Al-based energetic materials is not clear, which is a serious limitation for the application of oxides in PTFE/Al series energetic materials. In this paper, a variety of PTFE/Al/oxide energetic materials are fabricated with the addition of Bi_2_O_3_ and MoO_3_, respectively, with an oxide content ranging from 10% to 40% at 10% intervals. The energy release performance of PTFE/Al/oxide is investigated by ballistic impact experiments. Further, the enhancement mechanism of oxide on shock-induced energy release is revealed.

## 2. Materials and Methods

### 2.1. Sample Preparation

In this work, PTFE/Al/oxide series samples with different formulations were fabricated by PTFE powder, Al powder, Bi_2_O_3_ powder, and MoO_3_ powder. The physical and chemical properties of the raw materials are listed in Table 1.

PTFE, Bi_2_O_3_, and MoO_3_ as oxidants could, respectively, undergo violent redox reactions with the fuel Al, releasing a large amount of heat. The main combustion reaction could be expressed as follows:

(1) $${4\mathrm{Al}+3C}_{2}F_{4}{=4\mathrm{AlF}}_{3}+6C$$

(2) $${2\mathrm{Al}+\mathrm{Bi}}_{2}O_{3}{=\mathrm{Al}}_{2}O_{3}+3Bi$$

(3) $${2\mathrm{Al}+\mathrm{MoO}}_{3}={\mathrm{Al}}_{2}O_{3}+\mathrm{Mo}$$

The enthalpies of reactions, which are expressed by Equations (1)–(3), were, respectively, expressed as Δ_r_*H*_1_, Δ_r_*H*_2_, and Δ_r_*H*_3_, specifically −3435.4 kJ/mol, −1099.7 kJ/mol, and −930.2 kJ/mol. According to the above chemical reaction equations, the stoichiometry ratios of PTFE/Al, Al/Bi_2_O_3_, and Al/MoO_3_ were 73.5/26.5, 10.96/89.04, and 27.28/72.72, respectively. First, this work prepared the material formulation with PTFE/Al, Al/Bi_2_O_3_, and Al/MoO_3_ as independent units. Then, PTFE/Al Al/Bi_2_O_3_, and Al/MoO_3_ were mixed in different proportions to fabricate samples. The fundamental information of these samples studied in this work is listed in Table 2.

**Figure 1:**
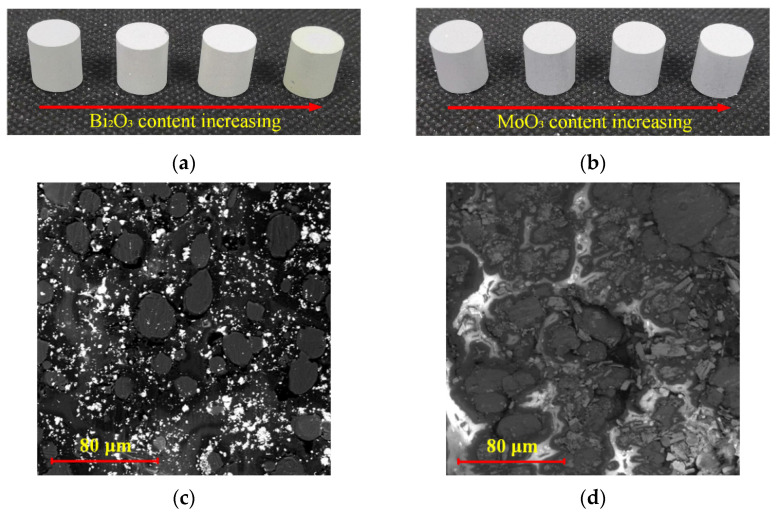
Typical sample (**a**) PTFE/Al/Bi_2_O_3_ with Bi_2_O_3_ content ranging from 10% to 40%; (**b**) PTFE/Al/MoO_3_ with MoO_3_ content ranging from 10% to 40%; (**c**) SEM of PTFE/Al/Bi_2_O_3_ (57.10/22.90/20); (**d**) SEM of PTFE/Al/MoO_3_ (53.29/26.71/20).

The preparation process of samples mainly included powder mixtures, cold pressing, and high-temperature sintering. Firstly, the raw powders (PTFE, Al, Bi_2_O_3_, or MoO_3_) of a certain mass were added to the anhydrous ethanol solution and mixed by a blender for approximately 60 min, followed by a drying process at room temperature lasting 48 h. Then, the mixed powder was placed in a mold with an inner diameter of 10 mm and cold uniaxial pressed at about 250 MPa. The designed size of the pressed sample was Φ 10 mm × 10 mm. Finally, samples after cold pressing were placed in a vacuum sintering oven. The oven temperature was raised to 370 °C at a rate of 50 °C/h, then stayed at 370 °C for 6 h and left to cool to ambient temperature naturally. The typical samples with different formulations are shown in Figure 1. As shown in Figure 1a, with the content of Bi_2_O_3_ increasing, the color of the sample gradually changed from gray to gray–green. As shown in Figure 1b, with the content of MoO_3_ increasing, the color of the sample remained gray–white. The morphology of the sample was strongly dependent on the morphology of oxide. The microstructure of typical samples is shown in Figure 1c,d. Al particles and oxide particles in the sample were uniformly wrapped in PTFE matrix.

### 2.2. Experimental Setup

**Figure 2:**
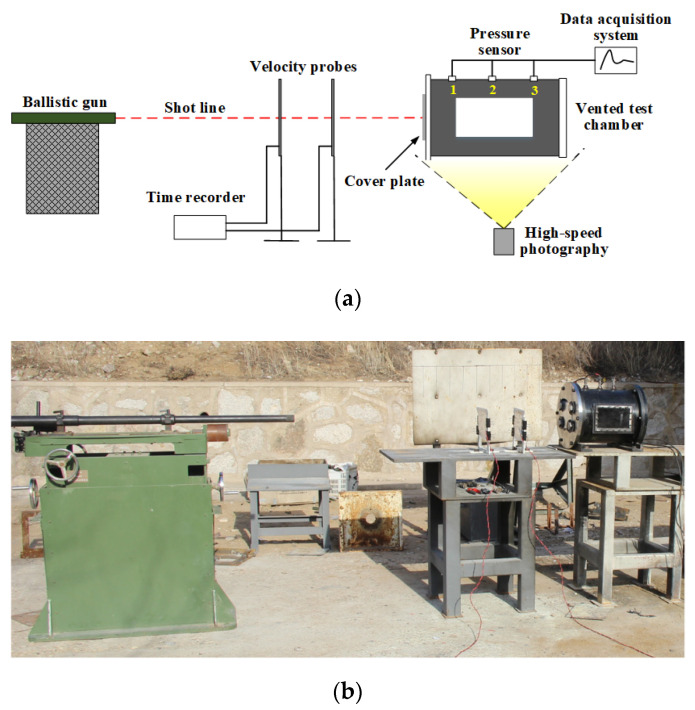
Schematic and physical photograph of experimental setup: (**a**) schematic; (**b**) physical photograph.

Figure 2 presents the schematic and physical photograph of experimental setup for studying the shock-induced energy releasing characteristics of PTFE/Al/oxide. The experimental system mainly consisted of ballistic gun, vented test chamber, pressure data measurement and acquisition system, and high-speed photography. The samples encapsulated in nylon sabots were launched from the 12.7 mm ballistic gun at the velocity of approximate 940 m/s. The 2024-T3 aluminum plates with thicknesses of 3 mm were used as the front cover plate of the vented test chamber with a volume of 27.35 L. The vented test chamber design was similar to the Ames design [14].

The pressure data measurement and acquisition system was the most important part of this experimental setup, including pressure sensors and the data acquisition system. The pressure sensor sampling frequency was 10 kHz with a measuring range of 0–1 MPa. The three pressure sensors were equidistant along the axis of the vented test chamber on the cylinder surface of the vented test chamber to measure the pressure caused by the energy release of the sample in the vented test chamber after impact. Because sensors 1# and 3# were close to the vent and the anvil in the chamber, respectively, the pressure data collected by sensor 2# were mainly adopted. The data from sensors 1# and 3# were used to confirm the authenticity of data measurements.

The Phantom V710 high-speed photography device (Vision Research, Inc., Wayne, NJ, USA) was employed to record the experiments. The selected frame rate was 20,000 per second, so that a frame was taken every 50 μs. The resolution was 640 × 480 pixels and the exposure time was set to 10 μs. These settings were selected based on early testing and represent an optimal tradeoff between available lighting and the minimization of blur in the images.

## 3. Results

**Figure 3:**
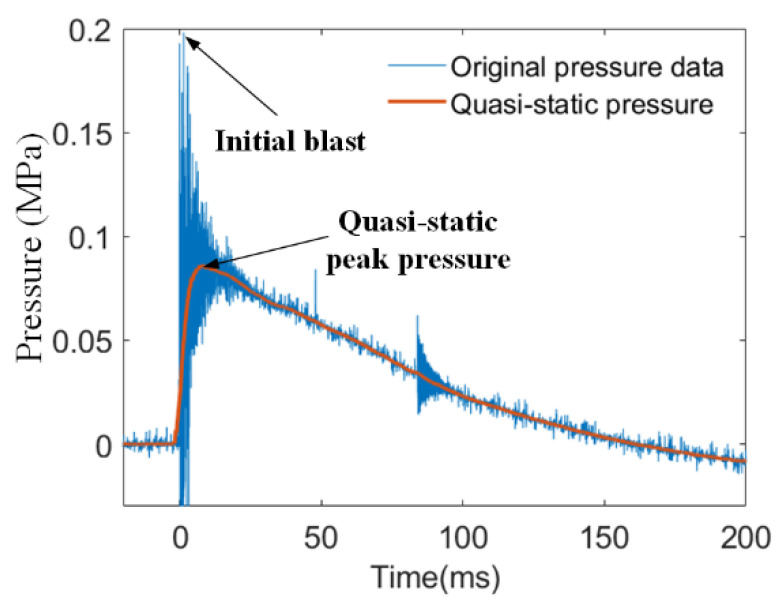
Typical original pressure data and quasi-static peak pressure.

The pressure variations which were recorded by the sensor directly reflected the energy release performance of PTFE/Al/oxide in the test chamber. As shown in Figure 3, the original pressure data indicated an extremely high peak at the initial stage of the reaction, followed by a sudden drop in pressure that gradually approached atmospheric pressure. The extremely high peak was usually several atmospheres high, caused by the initial detonation-like event of PTFE/Al/oxide. As shown in Figure 3, the initial pressure data were smoothed to obtain the quasi-static pressure data, which more effectively reflected the energy release of the energetic material in the test chamber.

**Figure 4:**
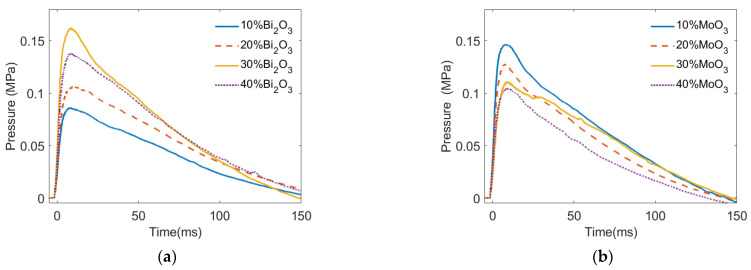
The pressure varied with time in the test chamber: (**a**) PTFE/Al/Bi_2_O_3_; (**b**) PTFE/Al/MoO_3_.

Figure 4 presents the quasi-static pressure of PTFE/Al/oxide as a generator varying with time. As shown in Figure 4, when the sample violently reacted in the vented test chamber after impact, the pressure in the vented test chamber increased rapidly to the quasi-static pressure peak in tens of microseconds, and then the pressure dropped to atmospheric pressure within hundreds of microseconds. In addition, it can also be seen from Figure 4 that the pressure variation induced by the deflagration of the PTFE/Al/oxide energetic material in the test chamber was affected by both the type and content of the oxide.

The pressurization characteristics of PTFE/Al/oxide are listed in Table 3. The quasi-static pressure peak (*P*_max_), impulse, and duration time were used to characterize the pressurization and energy release performance of the PTFE/Al/oxide energetic materials. The experiments showed that when the impact velocity ranged from 904 m/s to 971 m/s, the pressure variation characteristics of PTFE/Al/oxide varied with the oxide content. In addition, the influence of the oxide content on the pressurization characteristics of the PTFE/Al/oxide energetic material strongly depended on the oxide type. 

**Figure 5:**
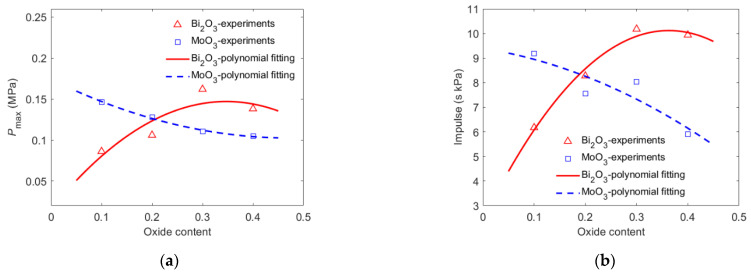
Pressure characteristics vary with content: (**a**) pressure peak varying with oxide content; (**b**) impulse varying with oxide content.

Figure 5 indicates that the pressure characteristics were strongly influenced by the oxide content and type. As shown in Figure 5a, when the content of Bi_2_O_3_ increased from 10% to 40%, the quasi pressure peak in the test chamber first increased and then decreased, from 0.086 MPa (10% Bi_2_O_3_) to 0.162 MPa (30% Bi_2_O_3_) and then decreased to 0.138 MPa (40% Bi_2_O_3_). The highest pressure peak of PTFE/Al/Bi_2_O_3_ was 1.88 times the lowest pressure peak. Moreover, when the MoO_3_ content increased from 10% to 40%, the pressure peak in the test chamber continued to decrease from 0.147 MPa (10% MoO_3_) to 0.105 MP (40% MoO_3_). The reaction of PTFE/Al/MoO_3_ with different oxide contents produced the highest pressure peak, 1.40 times the lowest pressure peak. It could be inferred that the optimal MoO_3_ content for PTFE/Al/MoO_3_ energy release was not within the range of 10% to 40%.

As shown in Figure 5b, the effect of the oxide content on the impulse of the PTFE/Al/oxide energetic material reaction was similar to that of the quasi-static pressure peak. With the increase in the Bi_2_O_3_ content from 10% to 40%, the impulse of PTFE/Al/Bi_2_O_3_ changed greatly, increasing from 6.175 s·kPa (Bi_2_O_3_ 10%) to the maximum value of 10.18 s·kPa (Bi_2_O_3_ 30%) and then decreasing to 9.940 s·kPa (Bi_2_O_3_ 40%). With the increase in the content of MoO_3_ from 10% to 40%, the PTFE/Al/MoO_3_ impulse decreased from 9.18 s·kPa (MoO_3_ 10%) to 5.91 s·kPa (MoO_3_ 40%). When the impact velocity was approximately 940 m/s, the effect of the Bi_2_O_3_ content on the pressure impulse of the PTFE/Al/oxide energetic material was greater than that of the MoO_3_ content. The content of Bi_2_O_3_ and MoO_3_ in PTFE/Al/oxide with the highest impulse was approximately 30% and less than 10%, respectively.

The preliminary analysis showed that in the PTFE/Al/oxide energetic material system, there were two reaction systems: PTFE/Al and Al/oxide. Among them, the PTFE/Al reaction had the characteristics of a lower ignition reaction temperature, slower burning rate, and longer reaction delay time. The characteristics of the Al/oxide reaction strongly depended on the properties of the oxide. The ignition temperatures of Al/MoO_3_ and Al/Bi_2_O_3_ were roughly the same, but compared to Al/MoO_3_, the Al/Bi_2_O_3_ mixture had the characteristics of a faster burning rate, a shorter delay time and a faster rate of pressure rise [14]. For PTFE/Al/oxide, as a mixture of energetic materials, the reaction characteristics depended on that of PTFE/Al and Al/oxide.

## 4. Discussion

### 4.1. Typical Shock-Induced Energy Release Behavior

**Figure 6:**
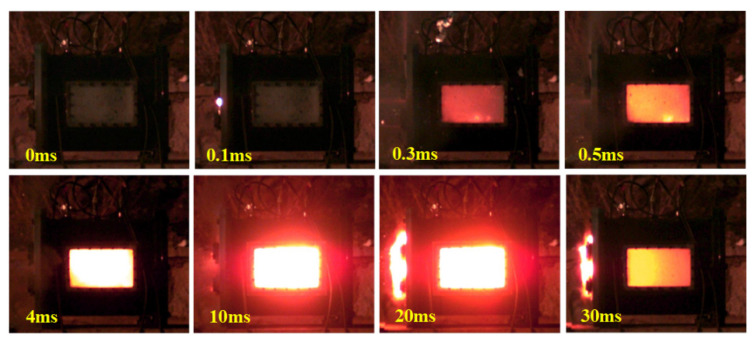
Typical frames of PTFE/Al/oxide (30% Bi_2_O_3_, *v* = 944.3 m/s) energy release process.

The findings and their implications should be discussed in the broadest context possible. Future research directions may also be highlighted. When the energetic material impact occurred, a shock wave was generated in the reactive material. Under the shock wave compression, the temperature of the energetic materials rose, inducing violent deflagrations. The deflagration of the PTFE/Al/oxide energetic materials released a large amount of heat, resulting in an increased pressure in the vented test chamber. As shown in Figure 6, the energy release behavior of the PTFE/Al/oxide energetic materials with an impact could be mainly divided into three stages. In the first stage, the PTFE/Al/oxide energetic material impacted the cover plate and shock waves were generated from the impact interface, which propagated from the head of the sample to the tail. The reaction was triggered immediately due to the highest shock wave (as shown in sequence 0.1 ms). In the second stage, the energetic material penetrated the cover plate and entered the test chamber (as shown in sequence 0.3 ms). With the propagation of the shock wave, the energetic material began to gradually react in the test chamber under shock wave compression and moved to the anvil. In the third stage, the energetic material was reignited (as shown in sequence 0.5 ms). The energetic material impacted with the anvil at the remaining velocity, causing the sample to splinter completely. After a few milliseconds, the reaction gradually stopped (as shown in sequence 30 ms). The gas, which originally existed in the test chamber, was rapidly heated by the reaction, which caused the pressure in the test chamber to rise rapidly, and the gas in the test chamber was discharged outwards under high pressure.

The relationship between the quasi-static pressure peak inside the test chamber and the energy released by the energetic material could be described as [15]

(4) $$\Delta E=\frac{V}{\gamma -1}P_{\max}$$

where Δ*E* is the energy release in the experiment, *P*_max_ is the quasi-static pressure peak, *V* is the volume of the test chamber, and *γ* is the ratio of specific heats of the gas (*γ* = 1.4). The energy release efficiency of PTFE/Al/oxide could be expressed as

(5) $$\eta =\frac{\Delta E}{\Delta E_{\max}}$$

where *η* is the energy released efficiency and Δ*E*_max_ is the theoretical chemical energy, respectively. Δ*E*_max_ depends on the content of Al/PTFE and Al/oxide in the sample, and the theoretical calculation could be expressed as

(6) $$\Delta E_{\max}=x_{\mathrm{Al}/\mathrm{PTFE}}e_{\mathrm{Al}/\mathrm{PTFE}}+x_{\mathrm{Al}/\mathrm{oxide}}e_{\mathrm{Al}/\mathrm{oxide}}$$

where *x*_Al/PTFE_ and *x*_Al/oxide_ are amounts of mass fraction of the reactants of two reactions, and *e*_Al/PTFE_ and *e*_Al/oxide_ are the theory of a unit mass containing chemical energy, which are calculated according to chemical reaction equations, such as Equations (1)–(3).

**Figure 7:**
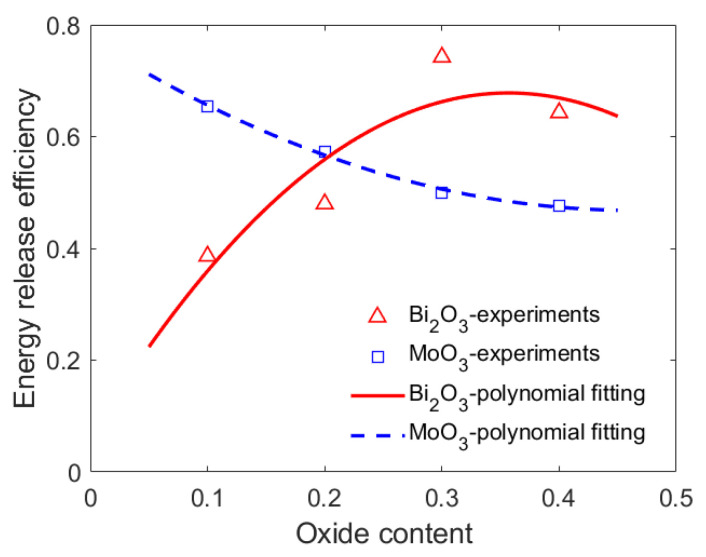
Energy release efficiency varying with oxide content.

In combination with Equations (4) and (5), the energy release efficiency of the PTFE/Al/oxide energetic material in the experiments was calculated, as shown in Figure 7. The effect of the oxide content on the energy release efficiencies of the PTFE/Al/oxide energetic materials described in Figure 7 was similar to that of the quasi pressure peak.

### 4.2. Shock Pressure Distribution of the PTFE/Al/Oxide

When PTFE/Al/oxide impacted the cover plate, the shock wave was generated and propagated within the energetic projectile. According to the one-dimensional shock wave theory [16], with the sample impact velocity *v*, the shock waves and particle velocities at the impact interface could be described as

(7) $$P_{s}=P_{c}$$

(8) $$v-u_{s}=u_{c}$$

where *P_s_* and *P_c_* are the shock wave generated in the sample and the cover plate, and *u_s_* and *u_c_* are the particle velocity in the sample and the cover plate, respectively. According to the conservation of energy and momentum, it could be expressed as

(9) $$P_{s}=\rho_{0s}U_{s}u_{s}$$

(10) $$P_{c}=\rho_{0c}U_{c}u_{c}$$

where *U_s_* and *U_c_* are the shock wave velocity of the sample and the cover plate, and *ρ*_0*s*_ and *ρ*_0*c*_ are the initial density of the sample and the cover plate, respectively.

The relationship between the particle velocity and the shock wave velocity could be described as

(11) $$U_{s}=C_{s}+S_{s}u_{s}$$

(12) $$U_{c}=C_{c}+S_{c}u_{c}$$

where *C_s_*, *C_c_*, *S_s_*, and *S_c_* are the Hugoniot parameters of the sample and cover plate, respectively. With the PTFE/Al/oxide as the mixture, the Hugoniot parameters could be calculated by using the following equation.

(13) $$\left\{\begin{array}{c} C=\sum m_{i}c_{i}\\ S=\sum m_{i}S_{i} \end{array}\right.$$

where *i* is the number of each component in the sample and *m_i_* is the mass fraction of the component, respectively.

During the axial propagation of the sample, the shock pressure decreased exponentially with the propagation distance *x*. Therefore, the shock pressure *P*(*x*) at each position in the sample could be expressed as

(14) $$P(x)=P_{s}\exp(-\delta x)$$

where *x* is the distance between the impact interface and the sample position and *δ* is the constant related to the material property, *δ* = 0.038 mm^−1^ [17]. Taking the added oxide such as Bi_2_O_3_ as an example, when the impact velocity of the sample was 900 m/s, according to the above analysis and calculation, the initial shock pressure after impact increased from 5.85 GPa to 7.74 GPa as the Bi_2_O_3_ content in the PTFE/Al/Bi_2_O_3_ material increased from 10% to 40%. The material property parameters related to the shock pressure are listed in Table 4 [18,19,20]. The initial shock pressure of PTFE/Al/Bi_2_O_3_ increased with the increase in the oxide content.

### 4.3. Energy Release Efficiency of the PTFE/Al/Oxide

Under the shock wave, the temperature of the material rose rapidly, inducing the deflagration of PTFE/Al/oxide. Assuming that the material heating process is an adiabatic process, according to the laws of thermodynamics and Hugoniot curves, the material temperature *T*_1_ generated under the action of shock wave intensity *P* could be expressed as [20]

(15) $$\begin{array}{l} T_{1}=T_{0}\exp\left[\left(\frac{\gamma_{0}}{V_{0}}\right)\left(V_{0}-V_{1}\right)\right]+\frac{V_{0}-V_{1}}{2C_{V}}P+\\ \;\;\;\;\;\;\;\;\;\frac{\exp\left[\left(-\gamma_{0}/V_{0}\right)V_{1}\right]}{2C_{V}}\int_{V_{0}}^{V_{1}}P\exp\left(\frac{\gamma_{0}}{V_{0}}V\right)\left[2-\frac{\gamma_{0}}{V_{0}}\left(V_{0}-V\right)\right]dV \end{array}$$

where *T* is the temperature, *γ* is the Gruneisen coefficient, *V* is the specific volume, *C_v_* is the heat capacity at a constant volume, and *P* is the shock pressure, respectively. Subscript 0 represents the material parameters in the initial state, and 1 represents the parameters after the shock wave compression state.

For the PTFE/Al/oxide energetic material, it was assumed that the chemical reaction rate and time were linear. According to the reach of the Ortega research [21], the energy released efficiency *η* and temperature relationship of the energetic material with temperature *T* could be expressed as

(16) $$\frac{dT}{d\eta }=\frac{R_{u}T^{2}}{E_{a}}\left[\frac{1}{2\eta }-\frac{n\ln\left(1-\eta \right)+n-1}{n\left(1-\eta \right)\left[-\ln\left(1-\eta \right)\right]}\right]$$

where *R_u_* is the universal gas constant, *E_a_* is the apparent activation energy, and *n* is the coefficient related to boundary conditions and reaction mechanisms.

In the PTFE/Al/oxide reaction system, the reaction mechanism was different from the reaction mechanism of Al/PTFE and Al/oxide reactions alone, so the coefficient *n* reflecting the PTFE/Al/oxide materials of the multi-reaction system could be estimated by the two reactions that can occur in the system.

(17) $$n=x_{\mathrm{Al}/\mathrm{PTFE}}n_{\mathrm{Al}/\mathrm{PTFE}}+x_{\mathrm{Al}/\mathrm{oxide}}n_{\mathrm{Al}/\mathrm{oxide}}$$

where *n*_Al/PTFE_ and *n*_Al/oxide_ are coefficients of two reactions, 0.625 [22] and 0.1 [23], respectively. Due to the similar reaction characteristics of thermite, the reaction coefficient of CuO was used to approximately replace the reaction coefficient of Al/oxide for the qualitative analysis. The reaction of the PTFE/Al/oxide system started from the Al/PTFE reaction with a lower activation energy [15], so the activation energy of the PTFE/Al/oxide material could be approximately considered as the Al/PTFE reaction activation energy, which was 50.836 kJ mol^−1^ [22].

**Figure 8:**
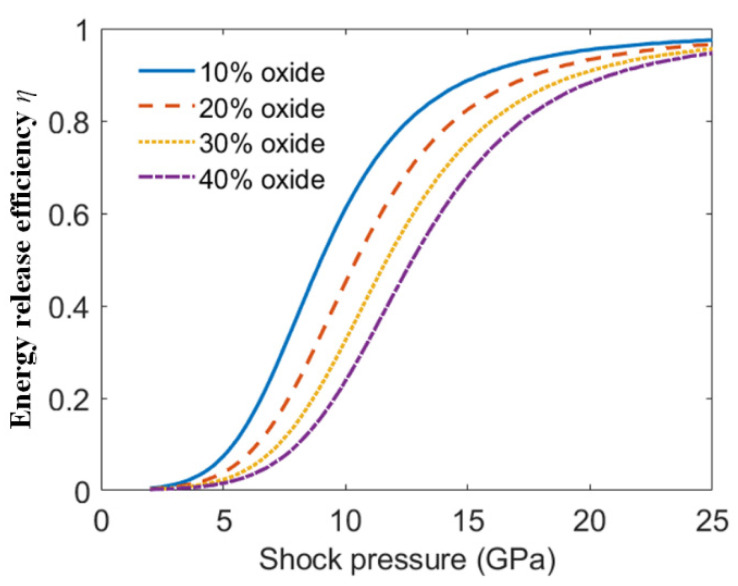
Energy release efficiency varying with shock pressure.

Combined with Equations (15)–(17), the reaction efficiencies varied with shock pressure in the PTFE/Al/oxide energetic material with a different oxide content, as shown in Figure 8. Figure 8 shows that when the energetic materials were subjected to the same intensity of shock wave, with the oxide content increasing, the energy release efficiency of PTFE/Al/oxide decreased.

**Figure 9:**
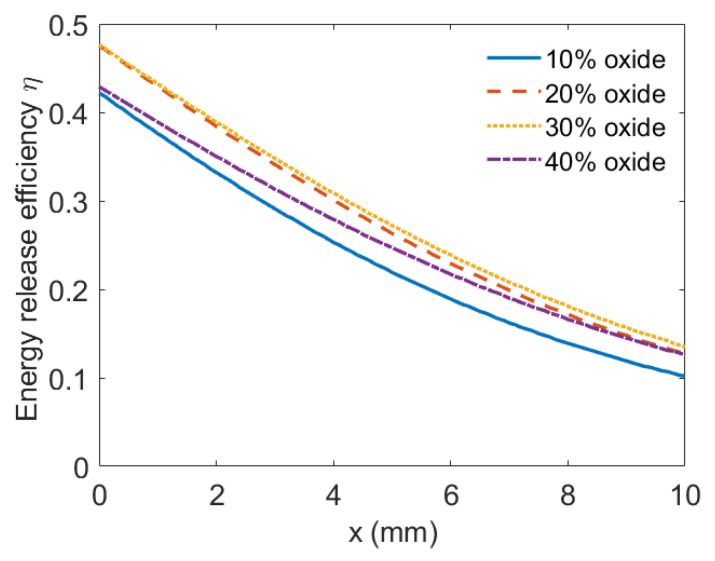
Energy release efficiency at each position of energetic materials.

The above analysis shows that the oxide content mainly affected the energy release performance of the PTFE/Al/oxide energetic materials from two aspects. On the one hand, due to the Bi_2_O_3_ content increasing, the initial shock pressure increased, which was conducive to the high temperature generated by the shock pressure compression. On the other hand, with the oxide content increasing, the chemical reaction extent of the energetic material decreased under the corresponding intensity due to the reaction mechanisms. For example, Figure 9 presents the energy release efficiency of the PTFE/Al-based energetic material with additional Bi_2_O_3_ at each position with the impact velocity of 940 m/s. As shown in Figure 9, as the distance from the impact head increased, the energy release efficiency of the energetic material decreased. In terms of the overall energy release efficiency, with the Bi_2_O_3_ content increasing from 10% to 40%, the energy release efficiency of PTFE/Al/Bi_2_O_3_ firstly increased and then decreased. Among them, the energy release efficiency was the highest when the Bi_2_O_3_ content was 30%. In addition, it is worth noting that the energy release efficiency calculated was lower than the experimental results, because the analytical model only considered the influence of the shock wave generated by the first impact. In essence, the energy release caused by the secondary impact was ignored. However, ignoring the above consideration would not have a great impact on the qualitative analysis. The analytical model could predict the influence of the Bi_2_O_3_ content on the energy release efficiency of the energetic material.

## 5. Conclusions

In this paper, the effect of the oxide content on the energy release performance of the PTFE/Al/oxide (oxide: Bi_2_O_3_ and MoO_3_) energetic materials was studied. The mechanism of the enhanced energy release performance of PTFE/Al/oxide materials was revealed, and the main conclusions were summarized as follows:

(1) With the Bi_2_O_3_ content increasing from 10% to 40%, the energy release of PTFE/Al/Bi_2_O_3_ increased first and then decreased. When the PTFE/Al had 30% Bi_2_O_3_ added, the pressure peak generated by the energetic material reached 0.162 MPa, which was 1.88 times higher than the one with 10% Bi_2_O_3_ (0.0862 MPa).
(2) The oxide content that caused the PTFE/Al/oxide release of maximum energy is called the optimal oxide content, and the optimal oxide content of PTFE/Al/Bi_2_O_3_ was approximately 30%.
(3) The PTFE/Al/oxide energy release mechanism analysis presented that the shock pressure and energy release efficiency under the corresponding shock pressure were affected by the oxide content, which depended on the energy release efficiency of PTFE/Al/oxide.
(4) With the same impact velocity, as the oxide content increased the shock pressure of PTFE/Al/oxide increased and the chemical reaction extent of the material under the corresponding shock wave intensity decreased.

## Figures and Tables

**Table 1:** Raw materials properties of samples.

Element	Manufacturer	Density (g·cm^−3^)	Measure Size (μm)	Shape	Enthalpy of Formation (kJ/mol)
PTFE	Donghu (Shanghai, China)	2.20	≈24	Sphere	−854
Al	Xingrongyuan (Beijing, China)	2.78	≈24	Sphere	0
Bi_2_O_3_	Xingrongyuan	8.90	≈38	Ovoid	−573.9
MoO_3_	Xingrongyuan	4.69	≈45	Random shape	−745.2

**Table 2:** Fundamental information of samples.

Material	Composition	Mass Fraction	TMD ^1^ (g/cm^3^)	TED ^2^ (kJ/g)
B1	PTFE/Al/Bi_2_O_3_	65.30/24.70/10	2.520	7.716
B2		57.10/22.90/20	2.744	7.013
B3		48.89/21.11/30	3.013	6.309
B4		40.69/19.31/40	3.341	5.606
M1	PTFE/Al/MoO_3_	63.39/26.61/10	2.468	7.908
M2		53.29/26.71/20	2.625	7.396
M3		43.18/26.82/30	2.803	6.885
M4		33.07/26.92/40	3.008	6.373

^1^ Theoretical maximum density. ^2^ Theoretical energy density.

**Table 3:** The reaction pressure characteristics of PTFE/Al/oxide in test chamber.

Material	*v* (m/s)	*P*_max_ (MPa)	Impulse (s kPa)	Duration (ms)
B1	970.1	0.086	6.175	144.54
B2	944.8	0.106	8.276	160.62
B3	940.3	0.162	10.177	139.52
B4	944.3	0.138	9.940	153.36
M1	959.0	0.147	9.184	134.50
M2	909.8	0.128	7.555	130.32
M3	970.9	0.111	8.034	137.66
M4	904.5	0.105	5.914	120.24

**Table 4:** The material property parameters related to the shock pressure.

Material	*ρ* (g cm^−3^)	*C* (m/s)	*S*	*C_v_* (J K^−1^)	*γ*
PTFE	2.20	1680	1.123	890	2.0
Al	2.78	5250	1.370	1020	0.6
Bi_2_O_3_	8.90	3432	0.275	235.7	0.796

## Data Availability

Not applicable.
